# Medical students’ and residents’ views on euthanasia

**DOI:** 10.1186/s12910-023-00986-x

**Published:** 2023-12-08

**Authors:** Rogério Aparecido Dedivitis, Leandro Luongo de Matos, Mario Augusto Ferrari de Castro, Andrea Anacleto Ferrari de Castro, Renata Rocha Giaxa, Patrícia Zen Tempski

**Affiliations:** 1 Department of Head and Neck Surgery, Hospital das Clínicas, University of São Paulo School of Medicine, São Paulo, Brazil; 2 Department of Head and Neck Surgery, Instituto do Câncer do Estado de São Paulo ICESP, University of São Paulo School of Medicine, São Paulo, Brazil; 3 Metropolitan University of Santos, Santos, Brazil; 4 Program of Postgraduation in Medical Sciences, UNIFOR and University of São Paulo School of medicine, São Paulo, Brazil; 5 Professor Health Education University of São Paulo School of medicine, São Paulo, Brazil; 6 Centro de Desenvolvimento de Ensino Médico – CEDEM, University of São Paulo School of Medicine, São Paulo, Brazil

**Keywords:** Euthanasia, Assisted dying, Medical students, Medical end-of-life decisions

## Abstract

**Background:**

Doctors are increasingly faced with end-of-life decisions. Little is known about how medical students approach euthanasia. The objective of this study was to evaluate, among medical students and residents, the view on euthanasia and its variants; correlate such a view with empathy and religiosity/spiritualism; and with the stages of medical training in Brazil.

**Methods:**

This is an exploratory cross-sectional study using an online questionnaire to be filled out on a voluntary basis among medical students and residents, consisting of: socio-demographic data, an empathy questionnaire and questions with elaborate clinical cases that typify situations of the variants of euthanasia.

**Results:**

From 1550 invitations, 273 volunteer participants responded (17.6%). The percentages of strong agreement/agreement on the concepts were: passive euthanasia (72.9%); active euthanasia (22.3%), orthothanasia (90.1%), dysthanasia (18.7%), assisted suicide (33%) and sedation (82.8%). Passive euthanasia, active euthanasia, dysthanasia and assisted suicide showed greater refusal with increasing length of medical training. Religious belief and degree of empathy did not significantly influence the opinion about the concepts. Strong agreement/agreement were: passive euthanasia (72.9%); active euthanasia (22.3%), orthothanasia (90.1%), dysthanasia (18.7%), assisted suicide (33%) and sedation (82.8%).

**Conclusions:**

Passive euthanasia, active euthanasia, dysthanasia and assisted suicide showed greater refusal with increasing length of medical training. The external validation of our findings relies on the distinct legal, cultural, and religious frameworks found across various countries.

**Supplementary Information:**

The online version contains supplementary material available at 10.1186/s12910-023-00986-x.

## Background

Physicians are increasingly faced with end-of-life decisions due to the aging process of the population and considerable advances in life-supporting and life-prolonging treatments, in which the dying process has already begun, and the prognosis is reserved. The bioethical principle of respect for patient autonomy is strongly present in the doctor-patient relationship in patients with incurable terminal illnesses [1].

Euthanasia means good death. It is applied to end, out of compassion, the life of someone suffering from an incurable disease, with unbearable pain and suffering. It represents the respect for the right to a dignified death in people who are seriously ill and whose will must be considered and involves the medical practitioner’s active termination of a patient’s life at the latter’s explicit request. It can be classified as: active (the medical practitioner initiates death by an action) and passive (by omission, when essential life-sustaining care is interrupted) [2].

Orthothanasia centers on upholding the dignity of a humane death, and viewing death as a natural progression marking the end of life. This approach permits patients to pass away naturally by discontinuing life-sustaining treatments deemed clinically futile, with the primary goal of alleviating suffering rather than hastening the end of life, even though it is understood that death will inevitably follow [2].

In physician-assisted suicide (autothanasia), the patient ends his or her own life, and may be assisted in doing so. This is the practice of providing a patient with a prescription for a drug that can be used with the primary intention of ending life. The purpose is to abbreviate irreversible pain and suffering.

Sedation in the terminal stages of life is the use of sedative drugs to reduce the level of consciousness and provide comfort for the patient until death occurs. In terminal sedation, the intent is to relieve intolerable suffering by means of sedative drugs for symptom control and relief of suffering. In contrast, in euthanasia, the intention is to kill the patient by administering a lethal drug and the successful result is immediate death [1].

With technology so effective in prolonging life, it is difficult to determine when it is appropriate to accept that a patient is dying, discontinue aggressive treatment, and strengthen palliative support. A number of issues contributes to the difficulty of withdrawing life-sustaining treatment: the distinction between stopping and interrupting treatment, religious and cultural considerations, the technological imperative, uncertainty of prognosis, variability in practice, and caregiver discomfort with death [3].

A total of 116 students at the University of Miami School of Medicine answered a questionnaire with simulated cases to evaluate decisions about allowing and helping patients who ask to die. The majority (78.9%) consider suspension of life support therapy consistent with passive euthanasia; passive euthanasia was considered murder for 3.5% and active euthanasia for 38%. Despite the fact that the majority (77.3%) agree that there is moral justification for helping patients die and feel understanding for a physician assisting a patient to die, only 6% would be willing to deliberately end a patient’s life by administering drugs to cause respiratory arrest [4].

Empathy is a cognitive quality that involves the ability to understand a patient’s experiences and perspectives. It is often associated with the quality of care in clinical practice and is particularly important among patients under end-of-life. In a holistic view of health, it is one of the central characteristics of medical professionalism and is often associated with quality of care in clinical practice as well as better health outcomes [5].

Religion and spirituality influence medical decision making in the case of terminal illness. Major religions vary dramatically in terms of their views on termination of treatment, euthanasia, pain control, and autopsy. Perspectives can vary even within the same religion based on the subgroup to which the patient belongs. The cultural practices and laws of a nation have a significant influence on beliefs and practices [6].

Many current medical ethics dilemmas will unfold, be discussed, and resolved, for better or worse, during the professional lives of medical students. Requests for assisted suicide and euthanasia are common in the everyday experiences of health professionals. Little is known about actual requests for student-directed assisted dying. It is not known how much students may become involved with such a scenario in and out of clinical settings. Little is also known about how medical students view euthanasia.

Thus, the objective of this study is to evaluate, by means of a voluntary questionnaire, among medical students and residents, the view on passive euthanasia, active euthanasia, orthothanasia, dysthanasia, assisted suicide, and sedation; to correlate such view with empathy and religiosity/spiritualism; and to correlate the findings with the different moments of medical training.

## Methods

### Study design

This is exploratory cross-sectional research. A survey questionnaire was developed for distribution to all medical students in each of the medical schools in Santos, Brazil, as well as to residents who work in the respective university-based hospitals, for voluntary completion. The study used an online survey with quantitative data to find out whether medical students and residents practicing in the city of Santos support, oppose, or are unsure about euthanasia and factors that support their views (available as a supplementary file).

### Sample recruitment

Students from the Faculdade de Ciências da Saúde da Universidade Metropolitana de Santos and Faculdade de Ciências Médicas de Santos da Fundação Lusíada, and residents from the Irmandade da Santa Casa da Misericórdia de Santos, Hospital Ana Costa, and Hospital Guilherme Álvaro were invited to participate. The sample included all medical students and residents in all years of study during the academic year 2021. The invitation to participate in the study was distributed via e-mail sent by the principal investigator and student with a directive position in the respective academic center in each medical school and by the medical residency committee of the hospitals.

### Measurements

The survey questionnaire consisted of: socio-demographic background of the respondent (including gender, age, highest level of education achieved by either parent, religion, frequency of religious practice); empathy questionnaire [5]; six multiple-choice questions with elaborate clinical cases that typify situations of passive euthanasia, active euthanasia, orthothanasia, dysthanasia, assisted suicide, and sedation, after due validation by a professional specialist in palliative care. The response options, for each case, were strongly agree; agree; disagree; and strongly disagree. The cases that typified each situation were previously validated by a palliative care specialist, and the participants were asked about the opinion adopted by fictitious physicians in each scenario.

### Statistical analysis

The comparison between groups was performed using the Kruskal-Wallis Test for independent samples, with a significance level of p < 0.05.

## Results

In total, 1550 invitations for participation were sent, with 273 voluntary participants (17.6%) responding. The sample consisted of 180 females (65.9%), distributed in a similar way among the different training periods. As for religious beliefs, most were Catholic (103 − 37.7%), followed by agnostics (84–30.8%), and Protestants and Kardecists (27 − 9.9% each). The majority did not attend religious services (140 − 51.3%)—Table 1.

The Jefferson Empathy Scale [5], which ranges from 70 to 140, had a mean score of 120.4, ranging from 90 to 140. The higher the score, the higher the degree of empathy.

**Table 1:** Respondent demographics

Variable	n = 273	n (%)
Gender	Male	93 (34.1)
	Female	180 (65.9)
Year in medical training	1st and 2nd years	59 (21.6)
	3rd e 4th years	60 (22)
	Internship	50 (18.3)
	R1/R2	51 (18.7)
	R3 a R5	53 (19.4)
Religion	Atheist	22 (8.1)
	Agnostic	84 (30.8)
	Catholic	103 (37.7)
	Protestant	27 (9.9)
	Kardecist	27 (9.9)
	Other	10 (3.7)
Attendance at religious services	Does not attend	140 (51.3)
	Annually	21 (7.7)
	6-Monthly	23 (8.4)
	Once every 1 to 6 months	45 (16.6)
	Fortnightly	12 (4.4)
	Weekly	32 (11.7)

In the series as a whole, the number and percentage of agreement/strong agreement was as follows: passive euthanasia (199 − 72.9%); active euthanasia (61–22.3%), orthothanasia (246 − 90.1%), dysthanasia (51–18.7%), assisted suicide (90 − 33%), and sedation (226 − 82.8%)—Table 2.

**Table 2:** Opinion regarding euthanasia and variants

Context	Strongly agree/agree	Neutral	Strongly disagree/disagree
Passive Euthanasia	199 (72.9%)	36 (13.2%)	38 (14%)
Active Euthanasia	61 (22.3%)	61 (22.3%)	181 (55.3%)
Orthothanasia	246 (90.1%)	12 (4.4%)	15 (5.5%)
Dysthanasia	51 (18.7%)	58 (21.2%)	164 (60%)
Assisted suicide	90 (33%)	69 (25.3%)	114 (41.7%)
Sedation	226 (82.8%)	32 (11.7%)	15 (5.5%)

**Fig. 1:**
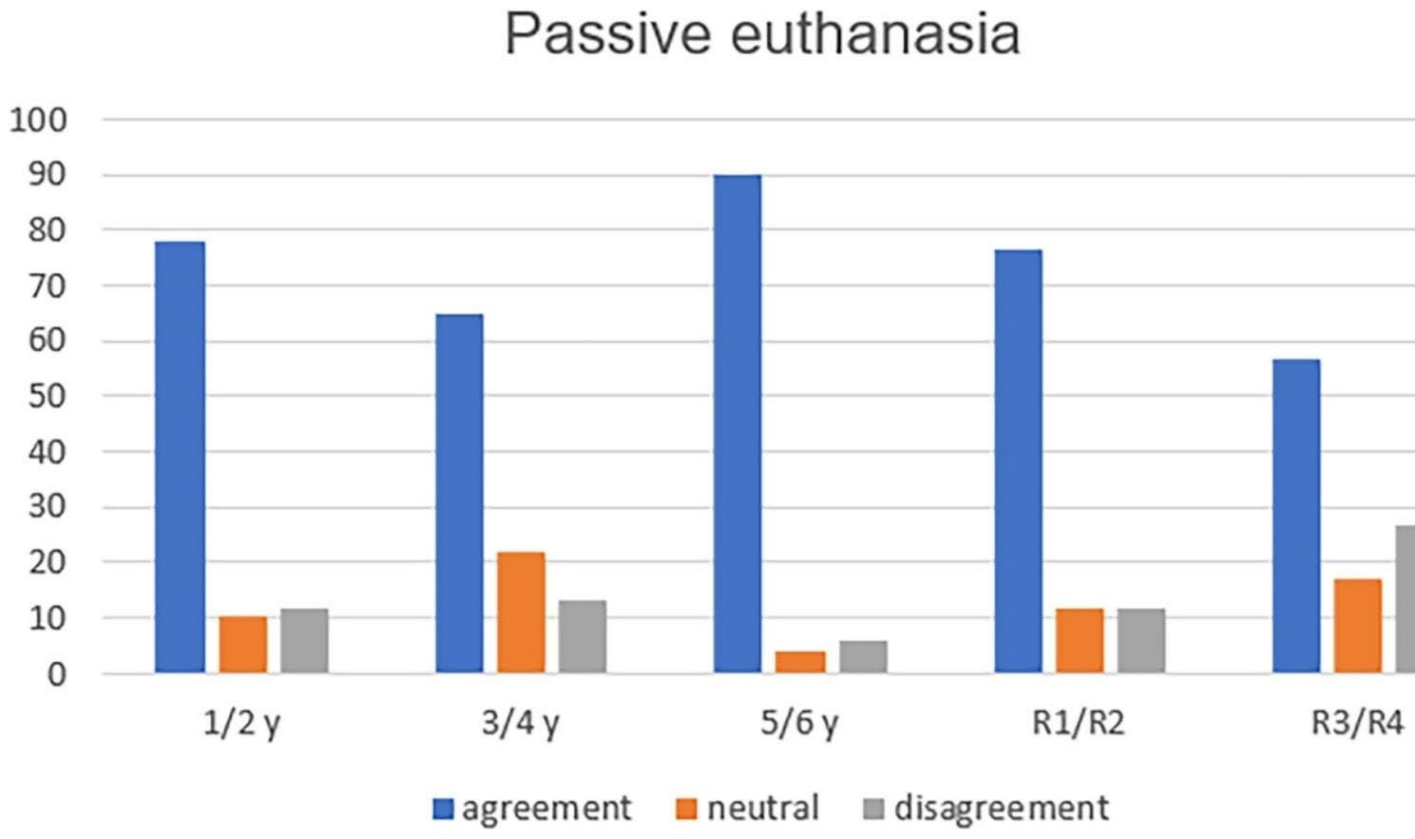
Opinion about the case of passive euthanasia throughout medical training (y = year of undergraduation; R1 till R4: from 1st to 4th year of medical residence)

**Fig. 2:**
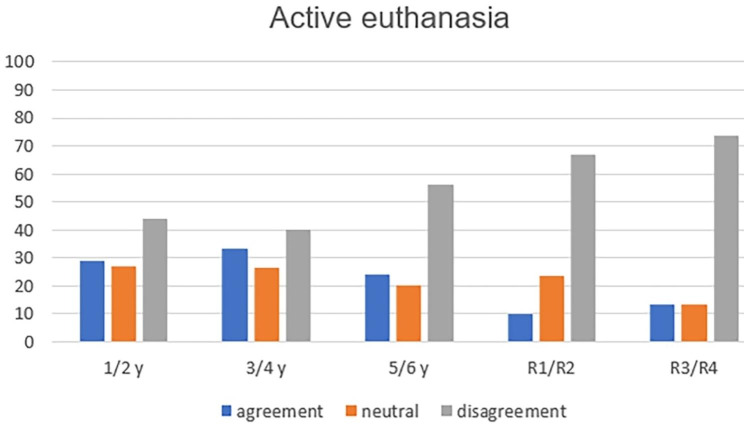
Opinion about the case of active euthanasia throughout medical training (y = year of undergraduation; R1 till R4: from 1st to 4th year of medical residence)

**Fig. 3:**
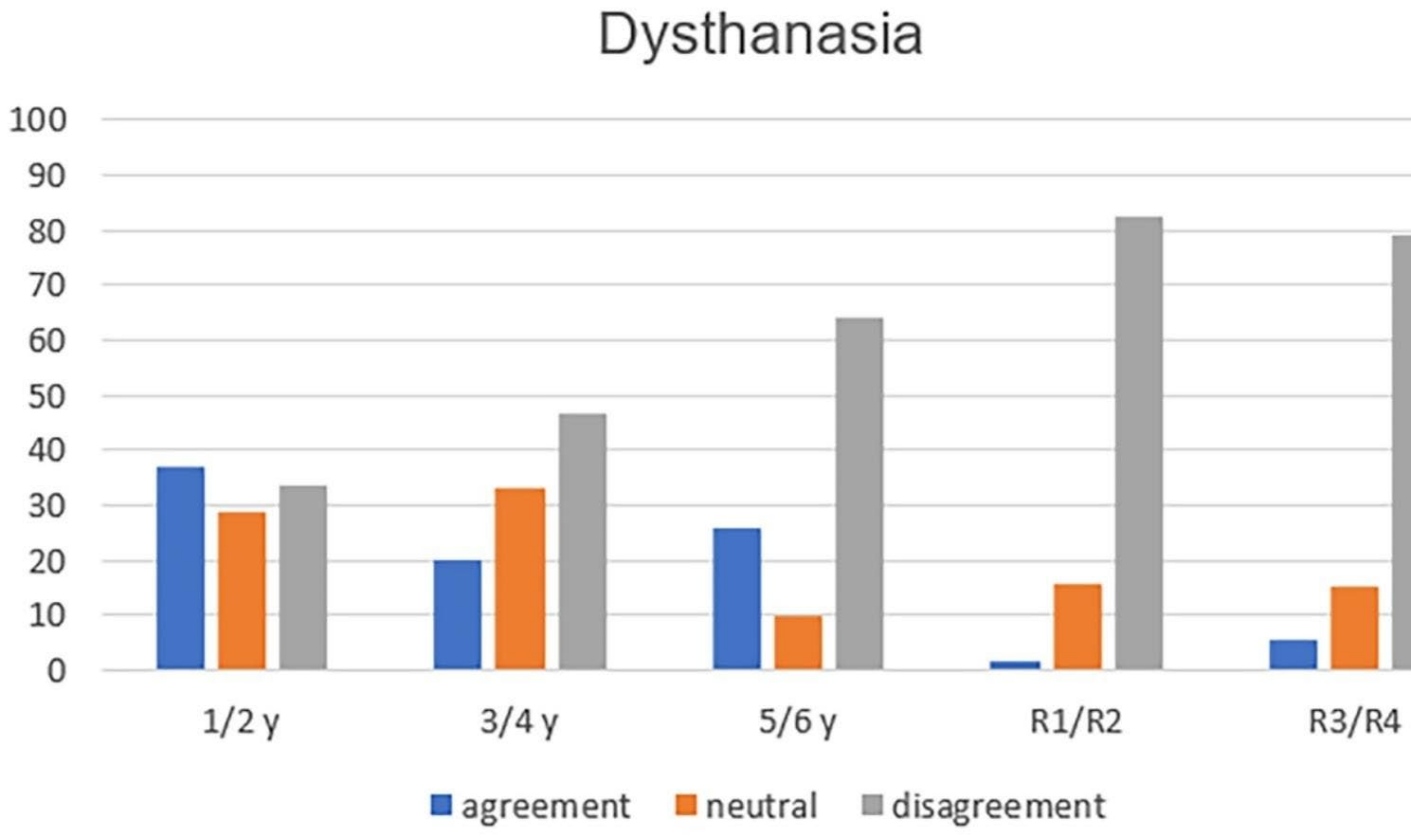
Opinion about the case of dysthanasia throughout medical training (y = year of undergraduation; R1 till R4: from 1st to 4th year of medical residence)

**Fig. 4:**
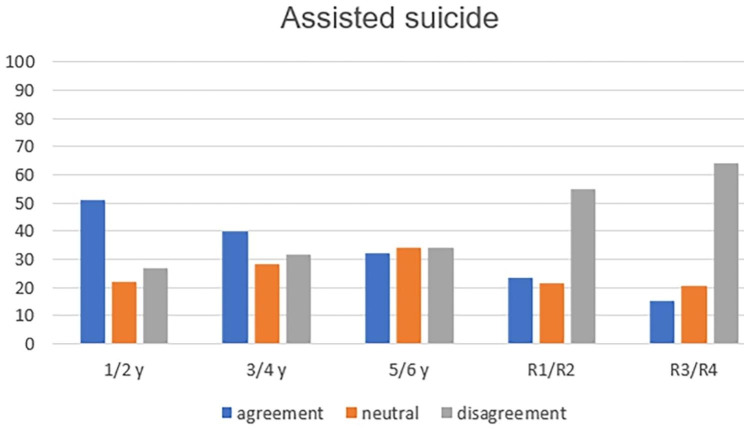
Opinion about the case of assisted suicide throughout medical training (y = year of undergraduation; R1 till R4: from 1st to 4th year of medical residence)

The analysis of the six concepts throughout medical training showed, with statistical significance, greater refusal to practice passive euthanasia (p = 0.004—Fig. 1); active euthanasia (p = 0.001—Fig. 2); dysthanasia (p < 0.001—Fig. 3); and assisted suicide (p = 0.005—Fig. 4).

The concepts were considered according to the respondents’ religious beliefs and the frequency with which they attended religious services, and no statistical difference was found. Similarly, the degree of empathy assessed by the Jefferson Scale [5] showed no significant impact on the respondents’ opinion with regard to the concepts.

## Discussion

In an anonymous survey at the University of New Mexico Medical School in Albuquerque, 96 residents (72% of respondents) participated. Residents were not inclined to perform these practices directly and did not support the conduct of assisted suicide practices by nonphysicians but were somewhat more accepting of other physicians’ involvement in assisted dying activities [7].

Attitudes toward active euthanasia and assisted suicide were surveyed among 520 medical, law, and psychology students at the University of Oslo, with a response rate of 59%. Considering medical students, 24% supported active euthanasia in cases of terminal illness and 35% supported assisted suicide. Among students of the Christian faith, 30% supported active euthanasia in terminal illness and 39% did so for assisted suicide. For students of no definable religion, the corresponding percentages was 65% [8].

A convenience sample of 372 physicians and 105 residents from York Health System community teaching hospital was surveyed anonymously. The response rate was 47%. Residents were less likely to support traditional prohibitions against physician-assisted suicide and voluntary active euthanasia and were more likely to offer assisted dying practices if they were legal. Residents were more likely to request both practices for themselves and family members [9].

A convenience sample of fifth- and sixth-year students (n = 160; response rate, 76.2%) at the University of Ferrara Medical School, Italy was studied; 28.3% responded that they were somewhat in favor or strongly in favor of euthanasia/assisted suicide (global), while 67.3% responded that they were somewhat in favor or strongly in favor of denying and/or suspending treatment for patients with incurable terminal illnesses; 36.9% somewhat or strongly agreed that fear of legal liability prevents oncologists from facilitating patient death [10].

At the University of New Mexico Medical School, of 306 students, 166 (54%) participated in an anonymous survey administered to medical school students. Respondents expressed opposition or uncertainty about assisted dying practices in cases of patients with severe forms of suffering secondary to amyotrophic lateral sclerosis, treatment-resistant depression and somatic disorders, antisocial and sexually violent behavior, or AIDS. They supported the withdrawal of life support through futile measures in AIDS patients [11].

By means of an anonymous questionnaire with different hypothetical scenarios concerning physician-assisted suicide, 140 medical students at the University of Fribourg, Switzerland were surveyed. Oncologists were more in favor of active euthanasia and physician-assisted suicide than palliative care specialists, but less in favor than medical students; 77% of students were in favor of active euthanasia and 70% were in favor of assisted suicide [12].

In a study at the Universidad Central del Caribe in Puerto Rico, 152 medical students, 62 resident physicians, and 84 members of three medical schools were questioned. 28% of students, 26% of residents, and 31% of faculty supported euthanasia; while 13% of students, 18% of residents, and 11% of faculty would engage in assisted suicide [13].

There has been an ongoing debate about a legalization of active euthanasia in Germany. An anonymous online questionnaire was sent to 1,092 third, fifth- and sixth-year students at two German universities: one with a mandatory (U1) and an optional (U2) palliative care subject. The response rate was 17.5%. Of all students, 21.1% in U1 and 37.2% in U2 could imagine performing active euthanasia on patients, although 72.6% in U1 and 78.2% in U2 thought its legalization would promote misuse. The main reasons were unbearable suffering and circumstances that lack dignity [14].

A questionnaire focused on end-of-life decisions was completed by 402 students at the Chinese University of Hong Kong. The number of students who felt that cardiopulmonary resuscitation should always be provided was higher in non-medical students (76/90, 84%) and medical students with less training (67/84, 80%) in year 1 vs. 18/67, 27% in year 5 (p < 0.001). Discontinuation of life support therapy was more accepted among senior medical students compared to junior medical students and non-medical students (27/66, 41%) in year 5 vs. 18/83 (22%) in year 1 and 20/90 (22%) among non-medical students (p = 0.003). Euthanasia was less accepted with more years of training (p < 0.001) [15].

A 20-item questionnaire was administered to medical students at the University of Athens, Greece. There were 251 participants, with 52% and 69.7% of the respondents being for the acceptance of euthanasia and physician-assisted suicide, respectively [16].

Attitudes toward euthanasia were assessed in 588 medical students at two universities in Poland. Four hundred ninety-two (84.97%) students were Catholic; 69 (11.73%) stated that they would practice euthanasia, 303 (51.53%) did not, and 216 students (36.73%) were not sure. The idea of legalizing euthanasia was supported by 174 (29.59%) respondents, against 277 (47.11%) and 137 (23.30%) were undecided [17].

In Mexico City, a questionnaire was answered by 140 residents at the beginning of their program and 99 third- or fourth-year medical students. There were no differences in acceptance between residents and students, but students from lay-run schools had significantly higher acceptance than students from religious schools for physician-assisted death (68 vs. 33%), termination of therapy (79 vs. 39%), and personalized assisted dying (57 vs. 48%) [18].

A cohort of 191 Connecticut physician assistants and 240 residents were evaluated on their willingness to follow the requests of the same elderly patients with decision-making capacity in five scenarios involving end-of-life care. Most assistants and residents were willing to comply with requests to maintain intubation (100% and 94%, respectively), to extubate (92% and 77%), and to give higher doses of narcotics (94% and 71%). Small proportions of assistants and residents were willing to prescribe a lethal number of sedatives (3% and 5%, respectively) and to give a lethal injection (1% and 4%), then illegal in that state. A significantly higher proportion of residents (32%) compared to assistants (19%) were willing to give a lethal injection, if it became legal [19].

Of 301 4th year medical students at the Technical University of Munich, 241 (80%) participated in a research. Most students were able to evaluate the legal norms on palliative sedation (legal) and euthanasia (illegal) correctly (81.2% and 93.7%, respectively), while only a few students knew that physician-assisted suicide at that time did not constitute a criminal offense; 83.3% of participants considered palliative sedation and the maintenance of artificial nutrition and hydration to be ethically acceptable, 51.2% considered physician-assisted suicide ethically legitimate, and 19.2% considered euthanasia ethically permissible [20].

An exploratory cross-sectional survey was distributed to medical students in a Canadian medical undergraduate program. There were 405 participants, with a response rate of 87%. Most students (88%) supported the Supreme Court decision striking down the ban on medical assistance in cases of death, 61% would provide the means for a patient to end his or her life, and 38% would personally administer a lethal drug. Students who were least willing to provide the means for such an assistance relied on religious/spiritual beliefs and teachings [21].

A cohort study was conducted using a self-completion survey questionnaire at a large UK medical school. In total, 400 of 505 questionnaires were completed (79%); 68.5% believed in God. Those who believed in God were more likely to disagree with actions that hasten death [22].

Medical assistance in dying became legal in Canada in 2016. Seventy-one preceptors and 62 residents were interviewed at Queen’s University in Ontario (membership, 45.2% of preceptors and 33.3% of residents). A low proportion of preceptors and residents felt competent or comfortable for discussing and exploring the situation with a patient [23].

A systematic review was conducted and 13 met the inclusion criteria. The highest acceptance rates for euthanasia and physician-assisted suicide were from European countries. The most common arguments supporting euthanasia and physician-assisted suicide were patient autonomy (n = 6), relief of suffering (n = 4), and the thought that terminally ill patients are additional burden (n = 2). The most common arguments against euthanasia were religious and personal beliefs (n = 4), the “slippery slope” argument and the risk of abuse (n = 4), and the physician’s role in preserving life (n = 2). Religion (n = 7), religiosity (n = 5), and attributes of the medical school of origin (n = 3) were the most significant variables in influencing students’ attitudes [24].

An online survey of undergraduate medical students at New Zealand’s Otago Medical School was conducted asking whether they supported a change in the law to allow euthanasia/assisted death. A total of 326 students responded to the survey (28% of those approached); 65% of 2nd year students supported euthanasia/assisted death, compared to 39% in 5th year [25].

Euthanasia, in the Brazilian Constitution, would be a privileging cause of homicide, while orthothanasia is a justification. Killing a terminally ill patient out of pity or compassion, at his request, for the abbreviation of unbearable physical suffering due to serious illness is considered a crime. However, stopping the maintenance of life by artificial means, provided death is certified as imminent or unavoidable, with the consent of the patient or his next of kin (orthothanasia) excludes unlawfulness. The process of death has already begun, and extraordinary procedures are suspended; it differs from passive euthanasia, in which procedures that could bring benefit to the patient are interrupted and such omission of care will be the motivation for death [2].

The approaches and reasons for statements on euthanasia in Brazil are revealed by the national legal provisions on the rights to health. According to the 1988 Constitution [26] (article 196), every citizen of Brazil has the right to health maintenance, which is guaranteed by the State through the implementation of the socioeconomic policy of universal and equal access to necessary medical services. On October 31st, 2018, the Brazilian Ministry of Health adopted Resolution 41: “On recommendations for the organization of palliative care within the scope of assistance provided by the Unified Health System” [27], which gave the provision of palliative care a regulatory format. Palliative care involves an open dialogue with the patient and their family about the goals of care, mainly aiming to preserve quality of life. A multidisciplinary team provides comprehensive care to people with potentially fatal diseases, from the moment of diagnosis until the end of the patient’s life, in order to alleviate the symptoms of the disease, especially physical pain [28]. The Code of Medical Ethics, approved by Resolution No. 1,931/2009 of the Federal Council of Medicine of Brazil [29], Art. 41, prohibits the doctor from shortening the patient’s life even at the request of the patient himself or his legal representative, and in its sole paragraph condemns dysthanasia for every patient who is in a terminal state. The Brazilian Penal Code [30] does not mention euthanasia, however, based on Art. 121, in the case of simple homicide, punishable by imprisonment from 6 to 20 years, the prison sentence can be reduced, in the terminology of Brazilian jurists, in so-called “pious” murders. Assisted suicide (suicide assistance) is included in art. 122 of the Brazilian Penal Code and is punishable by a prison sentence from 2 to 6 years if suicide occurred and from 1 to 3 years if the attempted suicide results in serious injuries. Although the Brazilian Penal Law is quite clear on the subject, from a moral point of view euthanasia is still controversial in Brazil.

The results presented may contribute important aspects in the education of medical students and residents, helping them to develop their future roles. There may be a lack of knowledge in this population about the possibility of relieving symptoms at the end of life. The symptoms listed as motivating euthanasia are in many cases possible to alleviate. Physicians’ attitudes about these issues are important in making decisions about end-of-life matters.

This study has some limitations, first and foremost, the low response rate – 273 voluntary responders out of 1,550 (17.6%). It did not cause the students and residents not to feel a sense of obligation and to provide free answers, however, possibly the cohort of responders could not fully represent the whole population studied. In fact in the literature the response rates varied from 47 to 87% [7–11, 20−22]. Second, our questionnaire was not validated separately. Finally, we did not include the responders’ self experience on caring for family members and loved ones, which could influence in their answers.

In summary, our study, along with previous research, reveals a range of attitudes and opinions on euthanasia and assisted suicide across different regions and within various demographic groups, with factors like religious beliefs, legal frameworks, and years of medical training potentially influencing these perspectives to some extent.

## Conclusions

The response rate of the questionnaires was 17.6% (273 out of 1,550). Among this population, strong agreement/agreement were passive euthanasia (72.9%); active euthanasia (22.3%), orthothanasia (90.1%), dysthanasia (18.7%), assisted suicide (33%) and sedation (82.8%). Passive euthanasia, active euthanasia, dysthanasia and assisted suicide showed greater refusal with increasing years of medical training. Religious belief and degree of empathy did not significantly influence the opinion about the concepts.

### Electronic supplementary material

Below is the link to the electronic supplementary material.

Supplementary Material 1: Online survey protocol.

## Data Availability

All the data are included on the manuscript.
