# Supplementary material for: Medical students’ and residents’ views on euthanasia
Source: BMC Med Ethics. 2023 Dec 8;24:109. doi: 10.1186/s12910-023-00986-x (PMC10704653; doi:10.1186/s12910-023-00986-x)
Supplement: Supplementary file 1 — Supplementary Material 1: Online survey protocol. [file 12910_2023_986_MOESM1_ESM.doc]

**Protocol**

**Parte I – Identification**

1. Gender: (1) man; (2) woman ................................................................... |___|

2. Age (years)> ..................................................................................... |___|___|

3. Where are you at in the medical training: (1) 1^st^ year; (2) 2^nd^ year; (3) 3^rd^ year; (4) 4^th^ year; (5) 5^th^ year; (6) 6^th^ year; (7) 1^st^ year of medical residence R1; (8) R2; (9) R3; (10) R4; (11) R5 ................................................................. |___|___|

4. Religion: (1) atheism; (2) agnostic; (3) Judaism; (4) Catholic: (5) Protestant; (6) Ortodox; (7) Islamism; (8) Kardecist; (9) African-based religion ............ |___|

5. Attendance at religious services: (1) does not attend; (2) annualy; (3) 6-monthly; (4) once every 1 to 3 months; (5) fortnighly; (6) weekly ............... |___|

**Passive Euthanasia**

Mr. Carlos, 66 years old, underwent a pneumectomy for cancer. He had chronic obstructive pulmonary disease and evolved postoperatively with respiratory failure, becoming dependent on assisted ventilation. During physiotherapy, he expressed his wish to die due to the poor quality of life conditions. He developed severe pneumonia and sepsis and was transferred to the ICU, where he was intubated and remained under mechanical ventilation and sedation. After 10 days, a tracheostomy was performed and sedation was removed; however, he remained in an irreversible coma and dependent on CPAP. Respecting Mr. Carlos' wish to die because of his suffering, Dr. Felipe, his physician for many years, decided to suspend the use of CPAP. After 10 hours, the patient died.

How do you interpret Dr. Felipe's conduct?

**Active Euthanasia**

In the same case of Mr. Carlos, still respecting his desire to die in the face of suffering, another doctor, Dr. Marcos, who had also been with him for many years, ended up taking over the case. Feeling great compassion for the patient, Dr. Marcos injected a lethal dose of medication and Mr. Carlos died.

How do you interpret Dr. Marcos' conduct?

**Orthothanasia**

Mr. Jose, 74 years old, underwent an esophagectomy for cancer. He had necrosis of the gastroesophageal anastomosis, with an enterocutaneous fistula. He underwent 10 subsequent operations without improvement of his condition. The chest wall was draining continuously, and he lost 20Kg in 6 months. During physical therapy, he said, despondently "I just want to die so I can rest". In control imaging examinations, recurrence of the disease in mediastinal lymph nodes and the presence of multiple liver metastases were detected in the PET-CT scan. The oncologist leading the case, Dr. Marcelo, ruled out any possibility of therapeutic salvage of the tumor. Mr. Jose developed pneumonia and was transferred to the ICU, was intubated, and remained on mechanical ventilation. Two days later, his wife and son agreed that DNR would be appropriate. Mr. José suffered cardiac arrest and the medical team did not perform resuscitation maneuvers. Mr. José died.

How do you interpret Dr. Marcelo's conduct?

**Dysthanasia**

Mr. Francisco, 70, underwent a pharyngolaryngectomy and neck dissection for cancer, followed by adjuvant radiotherapy and chemotherapy. He evolved with pharyngocutaneous fistula and had a massive cervical recurrence beyond treatment possibility. He lost 18 kg in 4 months. He continued to feed exclusively through a gastrostomy, and any possibility of reconstructing the alimentary transit for him to return to oral feeding was ruled out. Mr. Francisco developed pneumonia and was transferred to the ICU, and was intubated through a tracheostomy, remaining under mechanical ventilation. He evolved with septic shock, with the use of vasoactive drugs and refractory heart failure. His wife expressed to the doctor her wish that the patient could stay by her side, in the room, outside the ICU environment, in his last moments, but said she would follow the doctor's recommendation. Dr. Alfredo, the surgeon who has been with him since the onset of the condition, adopted as a conduct: start of prolonged parenteral nutrition, aiming to give better nutritional support; change of antibiotics, starting the use of latest generation drugs; and start of the use of extracorporeal membrane oxygenation (ECMO). Mr. Francisco remained under such care, in the ICU, in a coma, for another 7 days, and then evolved to death.

How do you interpret Dr. Alfredo's conduct?

**Assisted Suicide**

Mr. Cléber, 54, has a progressive degenerative neurological disease. At the moment he lives at home with a caregiver, requiring the use of a nasoenteral tube diet, tracheostomy care (performed for airway protection and better bronchial hygiene), and difficulty walking. The disease is considered irreversible, but with a slow course. During several consultations, he expressed his wish to die, because he understood he had a very poor quality of life. Dr. Victor has accompanied the patient for many years and has a great bond with him. Finally, he agrees to the administration by the patient himself, assisted by the doctor, of medication in a lethal dose. With Dr. Victor's assistance, Mr. Cleber injects the lethal solution and dies.

How do you interpret Dr. Victor's conduct?

**Sedation**

Mr. Alexandre, a successful 56-year-old businessperson, was diagnosed during routine examinations with gastric cancer and, at staging, presented with liver and bone metastases. Treatment with chemotherapy was established, but with no response. The patient presented progression of distant metastases, even after substitution with second-line drugs, and began to suffer from very intense pain. Even with the progression of analgesic measures, up to the use of morphine associated with other drugs, the patient maintained a very important and continuous pain. Once the possibilities of efficient analgesia were exhausted, and in compliance with the patient's express request, he resolved his personal and professional pending issues, said goodbye to his loved ones, and began the use of benzodiazepines with opioids administered intravenously. This reduced the level of consciousness and provided comfort to Mr. Alexandre who, after 5 days, evolved to death.

How do you interpret Dr. Artur's conduct?
